# *Astragalus mongholicus* Bunge and Panax notoginseng (Burkill) F.H. Chen Formula for Renal Injury in Diabetic Nephropathy—*In Vivo* and *In Vitro* Evidence for Autophagy Regulation

**DOI:** 10.3389/fphar.2020.00732

**Published:** 2020-06-12

**Authors:** Dan Wen, Rui-Zhi Tan, Chang-Ying Zhao, Jian-Chun Li, Xia Zhong, Hui Diao, Xiao Lin, Dayue Darrel Duan, Jun-Ming Fan, Xi-Sheng Xie, Li Wang

**Affiliations:** ^1^Research Center of Combined Traditional Chinese and Western Medicine, Affiliated Traditional Medicine Hospital, Southwest Medical University, Luzhou, China; ^2^Department of Nephrology, Affiliated Hospital of Southwest Medical University, Luzhou, China; ^3^Department of Endocrinology, Affiliated Traditional Medicine Hospital, Southwest Medical University, Luzhou, China; ^4^Center for Phenomics of Traditional Chinese Medicine, Southwest Medical University, Luzhou, China; ^5^Chengdu Medical College, Chengdu, China; ^6^Department of Nephrology, Nanchong Central Hospital, Nanchong, China

**Keywords:** *Astragalus mongholicus* Bunge and Panax notoginseng (Burkill) F.H. Chen formula, diabetic nephropathy, autophagy, mTOR, PINK1/Parkin

## Abstract

**Background:**

Diabetic nephropathy (DN) is a serious complication of diabetes mellitus (DM) with limited treatment options. DN leads to progressive renal failure and accelerates rapidly into end-stage renal disease. *Astragalus mongholicus* Bunge and Panax notoginseng (Burkill) F.H. Chen formula (APF) is a traditional Chinese medicine (TCM) formula widely used to treat chronic kidney diseases (CKD) in the clinic in the southwest of China. The aim of this study is to explore how APF and its related TCM theory work on DN and whether mTOR/PINK1/Parkin signaling plays a part in this process.

**Methods:**

HPLC was used for preliminary chemical analysis and quantitative analysis of the five components of APF. An *in vivo* autophagy deficiency model was established in C57BL/6 mice by streptozocin (STZ) combined with a high-fat and high-sugar diet, while the *in vitro* autophagy deficiency model was induced with high glucose (HG) in renal mesangial cells (RMCs). Renal histopathology staining was performed to investigate the extents of inflammation and injury. Real time-PCR and Western blotting techniques were utilized to assess autophagy-related proteins.

**Results:**

APF significantly ameliorated renal injury in DN mice, specifically restoring blood urea nitrogen, serum creatinine, and 24-hour albuminuria. APF also reduced the mRNA and protein expressions of TNFα, IL-1β, and IL-6 in STZ-induced DN mice. Furthermore, APF improved the autophagy deficiency induced by STZ *in vivo* or HG *in vitro*, as revealed by changes in the expressions of mTOR, PINK1, Parkin, Beclin 1, p62, and LC3B. Notably, inhibition of autophagy with 3-methyladenine in APF-treated RMCs aggravated cellular damage and altered mTOR/PINK1/Parkin signaling, indicating that APF rescued HG damage through promoting autophagy.

**Conclusion:**

APF may protect the kidneys from inflammation injuries in DN by upregulating autophagy *via* suppressing mTOR and activating PINK1/Parkin signaling. This experimental evidence strongly supports APF as a potential option for the prevention and treatment of DN.

**Graphical Abstract:**
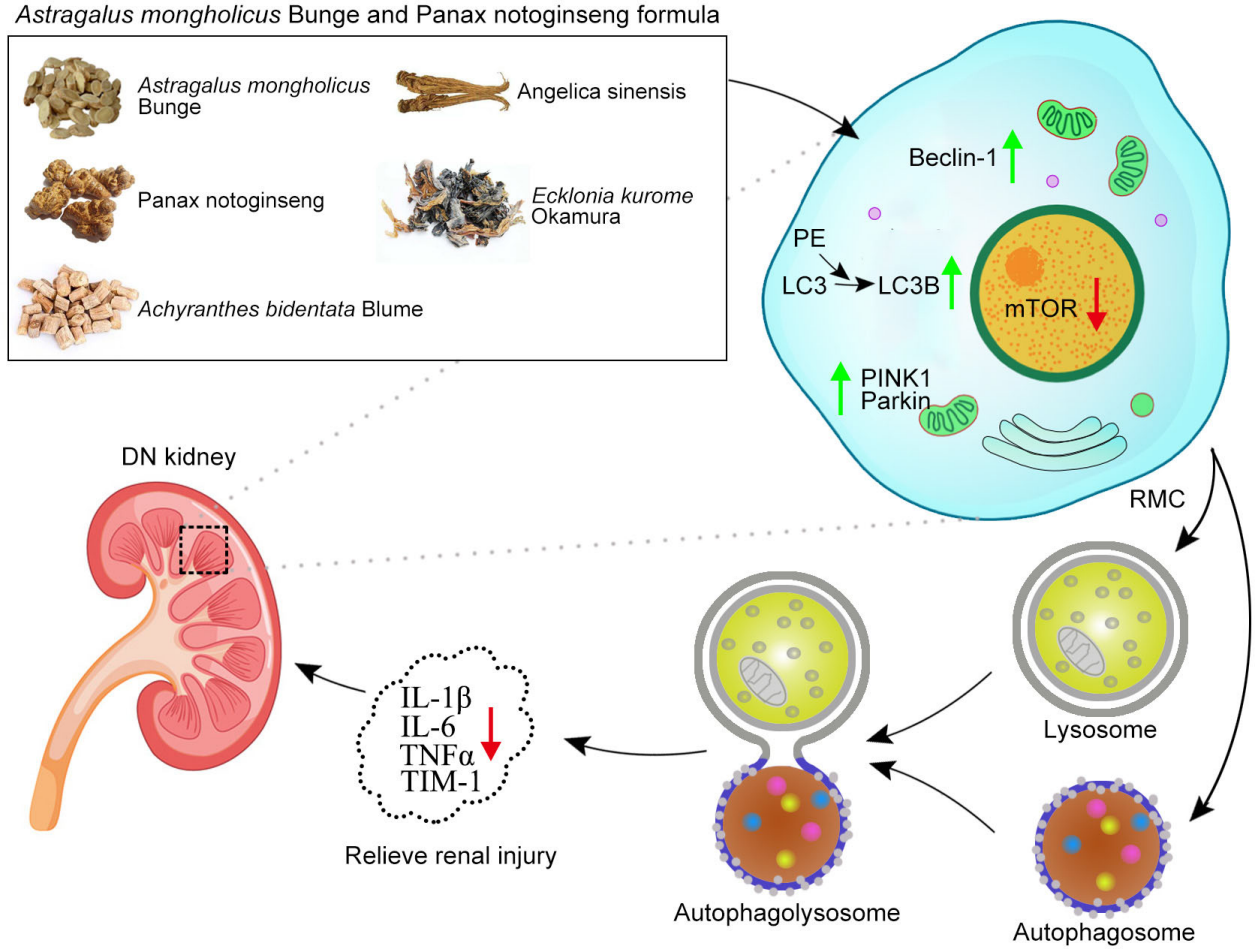


## Introduction

Diabetic nephropathy (DN) is a severe microangiopathic complication that occurs in about 35%~ 45% of type 2 diabetes mellitus (DM) patients and is attributed to 44% of the end-stage renal disease (ESRD) (Zheng et al., 2018). DN’s poor prognosis and high cost of treatment made it a serious risk to human health and a public health burden worldwide. Hypertension (Gangadhariah et al., 2015; Wysocki et al., 2017), hyperlipidemia (Mirzoyan et al., 2017), genetic predisposition (Gomes et al., 2012), and proteinuria (Maestroni and Zerbini, 2018) are known as risk factors for the occurrence or development of DN. Although symptomatic treatments have been applied to DN patients, including reducing hyperglycemia and controlling hypertension and intraglomerular pressure (Conway et al., 2012), which indeed have been proven to slow down the progression of DN to varying degrees, most patients still develop to ESRD. Therefore, further exploring the mechanism of the pathogenesis of DN and discovering new specific drugs continue to be important goals.

Over the years, emerging evidence has suggested that inflammation-related signaling pathways play a key role in the occurrence and development of DN (Navarro-González et al., 2011). Autophagy is a process in which damaged organelles are wrapped by autophagosomes and passed to the lysosome for intracellular degradation, thereby maintaining the homeostasis of the cells (Mizushima et al., 2011). In healthy bodies, macroautophagy/autophagy plays numerous roles in adjusting the microinflammation condition, and the regulation of inflammation through autophagy has great potential for treating renal injury. There is growing evidence that removing discarded organelles by autophagy protects kidneys from a variety of types of kidney inflammation, including acute, chronic, metabolic, and age-related inflammation (Mizushima and Komatsu, 2011; Kimura et al., 2017). Some studies proved that the expressions of the pro-inflammatory factors tumor necrosis factor α (TNFα), Interleukin-1 beta (IL-1β), and Interleukin-6 (IL-6) were significantly increased in high glucose (HG)-stimulated podocytes, while the level of heme oxygenase-1 (HO-1)-mediated autophagy prominently decreased. HO-1 functions as a beneficial enzyme mediating anti-inflammation and anti-oxidation through multiple pathways deriving mainly from macrophages (Nakamura et al., 2017; Zhan et al., 2018). Based on this phenomenon, we preliminarily hypothesized that autophagy could reduce the intracellular inflammation level by degrading intracellular wastes. It is worth mentioning that dysfunctions of autophagy and its related mammalian targets, rapamycin complex1 (mTORC1), AMP-activated kinase (AMPK), and Sirt1 in renal cells, like podocytes, tubular cells, and RMCs have been more or less proved to be related with the pathogenesis of multiple kidney diseases, including DN (Kitada et al., 2017). Moreover, hyperglycemia-induced mitochondrial injury mainly caused by excessive generation of reactive oxygen species (ROS) also largely reduced the function of autophagy, which may aggravate kidney injury in DN (Towler, 2013). Therefore, we believe there is sufficient evidence to prove that restoring autophagy helps to alleviate inflammation in kidneys, followed by improving the general condition of the kidney, which can play an important role in the occurrence and development of DN. This means that it may be an effective target for DN treatment.

Although substantial research has been conducted to illuminate the molecular mechanisms involved in DN, there are insufficient targets for treating this disease. It is worthy of notice that traditional Chinese herbal medicine has been widely used in Asia and is increasingly accepted by people in the West and has been widely used clinically (Li et al., 2011; Zhang et al., 2018). An increasing number of Chinese herbal formulas have been clinically applied, and remarkable results have been achieved. The *Astragalus mongholicus* Bunge and Panax notoginseng (Burkill) F.H. Chen formula (APF) consists of Astragalus mongholicus Bunge, Panax notoginseng (Burkill) F.H. Chen, Angelica sinensis (Oliv.) Diels, Achyranthes bidentata Blume, and Ecklonia kurome Okamura. Many years of practice and preliminary experiments in our research have proved that APF can significantly alleviate fibrosis in chronic kidney disease (CKD) (Wen et al., 2014), protect residual kidney function, have anti-inflammation, antioxidation, and anti-apoptosis effects, and improve the lives of patients in the most critical early stage of acute kidney injury (AKI). Among the five components mentioned above, *Astragalus mongholicus* Bunge and Panax notoginseng (Burkill) F.H. Chen were regarded as “JUN” medicines in traditional Chinese medicine (TCM) theory. “JUN” medicines are the drugs that play the fundamental function in a formula. *Astragalus mongholicus* Bunge, known as “Huangqi” in China, is a basic component of APF and can fight against fibrosis, enhance the immune function while reducing inflammation, and protect the liver from CCl4-induced necrosis (Hamid et al., 2017) as a result of its anti-oxidative (Kolbert et al., 2018), anti-hypertension (Xue et al., 2008), and anti-bacterial (Labed et al., 2016) properties. Modern medicine generally accepts that Panax notoginseng (Burkill) F.H. Chen, known as “Sanqi” and “Tianqi,” has a wide range of pharmacological effects on cardiovascular, endocrine, immune, and urinary systems, such as being anti-fibrosis, reducing swelling, relieving pain and hemostasis, being anti-thrombosis, and so on (Xia et al., 2011; Tian et al., 2019). The “CHEN” medicines Angelica sinensis (Oliv.) Diels and *Ecklonia kurome* Okamura were believed to assist the function of the “JUN” medicines. In the TCM prescription, the reasonable compatibility of many kinds of medicinal materials can increase effectiveness.

Astragaloside IV is the main component profile extracted from *Astragalus mongholicus* Bunge. It was proven of to have an anti-oxidant stress function and can inhibit the activity of TNFα, IL-1β, and IL-6 to achieve an anti-inflammation effect (Yu et al., 2016). Among the many standard ingredients that we successfully tested, Ferulic acid is one of the main ingredients extracted from Angelica sinensis (Oliv.) Diels, and its working mechanisms may be associated with its anti-apoptotic, anti-inflammatory, and anti-tumor action (Li et al., 2017; Wang et al., 2018).

However, whether APF can ameliorate the autophagy function of impaired kidney has not previously been discussed. Likewise, how APF works in the DN is not yet completely clear. Therefore, in this study, we applied gradient concentrations of APF to DN C57BL/6 mice to explore how APF functions in DN kidneys. For *in vivo* experiments, in addition to proving the redeeming effect of APF-containing serum on HG-induced renal mesangial cells (RMCs), we used 3-Methyladenine (3-MA) to reduce the autophagy level to explore whether APF could improve the damage caused by HG stimulation by regulating the autophagy level.

## Materials and Methods

### *Astragalus mongholicus* Bunge and Panax notoginseng (Burkill) F.H. Chen Formula (APF)

The APF used in this study is not a mixture of traditional Chinese medicine decoction pieces but a mixture of herbal extract granules extracted from Chinese herbal medicine by a specialized process in the Department of Pharmacy of the Traditional Chinese Medicine Hospital, affiliated to Southwest Medical University. The herbal extract granules extraction technique can completely extract the main component profile in traditional Chinese medicine decoction pieces through modern and efficient extraction technology. These granules can have the same efficacy as the traditional Chinese medicine decoction pieces while avoiding the troublesome preservation and decocting process. APF contains Panax notoginseng (Burkill) F.H. Chen (1 g), *Astragalus mongholicus* Bunge (3 g), Angelica sinensis (Oliv.) Diels (3 g), *Achyranthes bidentata* Blume (3 g), and *Ecklonia kurome* Okamura (3 g) with a total weight of 13 g. In the clinical use of APF, a daily dose for an adult is formulated as 13 g per day. According to the conversion ratio between mice and humans (9.1), the daily APF dose (g/kg) for mice can be calculated as the following formula: conversion ratio of surface area between mice and humans (9.1) × a daily dose of an adult (13 g)/average weight of adults (60 kg), that is: 9.1 × 13 (g)/60 (kg) = 1972 mg/kg. The minimum daily dose of mice was set as the low-dose intervention group, the medium-dose was twice this (3944 mg/kg), and the high-dose was four times this (7888 mg/kg). In order to better understand the effectiveness of this formula from the perspective of effective ingredients, HPLC technology was applied to detect the content of main component monomers that can be extracted from the APF as a whole.

### Chemicals and Reagents

Streptozotocin (Cat: ALX-380-010-G001, Lot: 04081408) was purchased from Enzo Life Sciences Inc. (New York, USA). Antibodies against p-mTOR (Cat: #2971, Lot: 21, rabbit anti-mouse), mTOR (Cat: #2983, Lot: 14, rabbit anti-mouse), Beclin1 (Cat: #3495, Lot: 6, rabbit anti-mouse), PINK1 (Cat: #6946, Lot: 4, rabbit anti-mouse), and LC3B (Cat: #3868, Lot: 13, rabbit anti-mouse) were purchased from Cell Signaling Technology, Inc. (Danvers, MA, USA). Antibodies against β-actin (Cat: ab8226, anti-mouse)), Parkin (Cat: ab77924, Lot: GR3186724-4, rabbit anti-mouse), kidney injury marker 1 (TIM1/KIM1) (Cat: ab47634, Lot: GR189759-26, polyclonal, rabbit anti-mouse), IL-6 (Cat: ab9324, Lot: GR237999-7, anti-mouse), and p62 (Cat: ab109012, Lot: GR3241806-3, anti-rabbit) were purchased from Abcam Technology (Cambridge, UK). IL-1β (Cat: sc-52012, Lot: #11817, anti-mouse) was purchased from Santa Cruz Biotechnology (Shanghai, China). Blood serum urea nitrogen (Cat: C013-2, Lot: 20180504), serum creatinine (Cat: C011-2, Lot: 20181205), and 24-h albuminuria assay kit (Cat: C035-2, Lot: 20180904) were purchased from Jiancheng Biologic Project Company (Nanjing, China). Triton-X (Cat: #H5141, Lot: #104355) was purchased from Sangon Biotech (Shanghai, China). 3-methyladenine (Cat: S2767) was purchased from Selleckchem (Houston, TX, USA). RNAsimple Total RNA Kit T and RT Reagent Kit (Cat: DP419, Lot: 5111) were acquired from Tiangen (Beijing, China). HE staining kit (Cat: C0105) was purchased from Beyotime Biotechnology (Beijing, China). PAS (Periodic Acid-Schiff stain) kit (Cat: G1281, Lot: 20190122) was purchased from Solerbio (Beijing, China). Masson kit (Cat: BA-4079B, Lot: 617111) was purchased from BASO company (Zhuhai, China). Immunohistochemical kit (Cat: PV-6000, Lot: 17G67D12) was purchased from ZSGB-BIO (Beijing, China). DAB kit (Cat: ZLI-9019, Lot: K183316C) was purchased from ZSGB-BIO (Beijing, China). CellTiter 96 AQueous One Solution Cell Proliferation Assay (CCK-8) (Cat: G3580) was purchased from Promega (Beijing, China). ECL (enhanced chemiluminescence) (Cat: 34580, Lot: TG268507) was purchased from Thermo Scientific (Massachusetts, USA). OCT (Cat: 4583, Lot: 7076-00) was purchased from Sakura Finetek USA. PVDF membranes (Cat: IPVH00010, Lot: K5NA8024C) was purchased from Immobilon-P (EMD Millipore Co., Billienca, USA). Mannitol (Lot: A18021306-2) was purchased from KELUN Industry Group (Chengdu, China). D-(+)-Glucose (Cat: G7021-100G, Lot: #SLBR0902V) was purchased from Sigma Aldrich (St. Louis, MO, USA). Fetal Bovine Serum (Cat: 10099-141C, Lot: 2094468CP) was purchased from Gibco BioTek (Massachusetts, USA). Basal DMED/LOW glucose medium (Cat: SH30021.01, Lot: AD14890264) was purchased from HyClone, Thermo Scientific (Massachusetts, USA).

### Preparation of APF-Containing Serum

Due to the need for purity of cell intervention drugs in *in vitro* experiments, we needed rats to absorb APF from stomach to blood. The serum containing APF was used in subsequent trials so as to protect RMCs from the interference of impurities in the herbal medicine. Male SD rats were administered by gavage with APF (1365 mg/kg) or normal saline (NS) once a day at the same time for three consecutive days. Two hours after the final gavage, the rats were anesthetized and sacrificed, and the blood was collected from the aortaventralis. The blood samples were saved overnight at 4°C and then centrifuged at 3000 rpm/min for 15 min. The upper serum was collected and incubated in a water bath at 56°C for 30 min for inactivation then freeze-dried with a freeze dryer (Biocool, Beijing, China) and stored at -80°C. The powder containing APF was dissolved in basal culture medium and filtrated through a 0.22-μm filter screen before being applied to RMCs.

### Cell Culture and Treatment

The RMCs were a gift from Prof. Lan at the Chinese University of Hong Kong. RMCs were cultured in basal culture medium containing 10% fetal bovine serum and 100 U/ml penicillin, 100 mg/ml streptomycin at 37°C in a 5% CO_2_ atmosphere. High-glucose (30mM) culture medium was made from a specific proportion of 65 mM D-(+)-glucose and basal culture medium.

### Animals Experiments

Eight-week-old male C57BL/6 mice with a weight of 20 ± 2 g were purchased from Chengdu Dossy Experimental Animal Co. Ltd. (Chengdu, China) and placed under adaptive feeding for a week in a temperature- and humidity-controlled environment (12-h light/dark cycle) with unlimited access to water and standard chow. Thirty-six mice were randomly and averagely divided into six groups as follows: blank control group (Ctrl group), model group (DN group), low/medium/high-APF prevention groups (DN+APF-L group, DN+APF-M group, and DN+APF-H group), and positive control group (DN+Irb group). The mice in the control group continued to receive standard chow as before, while the mice from the modeling and intervention groups were fed a high-fat and high-sugar diet for 8 weeks. After 8 weeks, the tails of all mice in the special diet groups were nicked to collect approximately 2 μL for blood glucose assay after fasting for over 12 hours, and the mice were regarded as successful DM models when their fasting blood glucose values were over 11.1 mM. Next, the model mice received intraperitoneal injections of streptozocin (STZ) at a dose of 50 mg/kg for five consecutive days to destroy the β-cells in their islets. Real-time detection of 24-h albuminuria of those models was then performed for over two weeks until it was more than 3.5 mg/day, at which point they were considered successful DN models. Subsequently, all DN mice were randomly divided into the following five groups: the vehicle control group, which received jelly with no medicine in it, the DN group, the low dose of APF prevention group (DN+APF-L, 1972mg/kg), the medium dose of APF prevention group (DN+APF-M, 3944mg/kg), the high dose of APF prevention group (DN+APF-H, 7888mg/kg), and the positive-control irbesartan prevention group (DN+Irb, 20 mg/kg). All mice in the model group and the treatment groups continued to be fed a high-fat and high-sugar diet during the 8-week APF intervention and were weighed once a week. At the end of the experiment, blood samples from the mice were collected from orbital veins. Hearts, livers, spleens, lungs, and kidneys of all mice were collected, and the tissues were stored in 10% formalin or -80 °C for later use. All animal experiments were conducted under the guidelines of the Animal Ethics Committee of Southwest Medical University.

### APF Administration

Mice in the treatment groups were fed with hand-made jelly containing medicine. Edible gelatin was dissolved in drinking water and microwaved on high for 3 min then mixed with powder of normal mouse feed and decoction of APF. Next, a 24-well plate was filled with the tepid mixture.

### CCK-8 Assays

The cell viability of RMCs was tested with Cell Counting Kit-8 (CCK-8, Promega) according to the manufacturer’s protocol. Briefly, RMCs were seeded in 96-well plates in each group for cell viability assay, respectively. CCK-8 reagent was dissolved in a tenfold volume of basal culture medium before adding 100 μL to each well. After approximately two hours, the IOD value of each was detected by microplate reader (Bio Tek Instruments, Inc, USA). The viability of RMCs with different treatment conditions was then calculated from their corresponding IOD values.

### Assessment of Renal Function and Physiological Function

Blood samples from mice were collected from orbital veins and centrifuged (3000 rpm/min, 10 min) before being placed at 4°C overnight. The next day, serum on the top was collected to perform blood urea nitrogen (BUN), serum creatinine (Scr), and 24-h UAlb determination. After 6 h of fasting, fasting blood glucose (FBG) was detected with blood glucose test strips (Omron, Japan), and glucose tolerance test (1.5 g/kg glucose i.p.) was performed on the mice (before being executed). Urine of mice was collected with a 24-h metabolism cage separately for 24-h albuminuria detection.

### HE Staining and PAS Staining

Fresh kidney tissues were fixed in a 10% formalin solution prepared with PBS for at least twenty-four hours and then embedded in paraffin before cutting into 4-μm slices. All sections are finally stained with hematoxylin to distinguish cell nuclei. Pathology sections for immunofluorescence, specifically, are embedded with OCT on liquid nitrogen and were cut at -20°C in a freezing microtome before passing on to the next part of the experimental program. All sections are also stained with hematoxylin to distinguish cell nuclei. All paraffin sections are stained with PAS reagent and hematoxylin-eosin (HE) reagent to identify kidney structure and also stained with Masson reagent to identify blue collagenous fiber. Pathology sections were immunohistochemical stained to reveal tumor necrosis factor α (TNFα), LC3B, and Parkin protein levels.

### Immunofluorescence Staining

Renal mesangial cells were seeded on cell slides and treated as detailed above for immunofluorescence. First, cell slides were fixed with 4% paraformaldehyde and were dehydrated with graded ethanol before being embedded in OCT. Subsequently, 4-μm sections were incubated with primary rabbit anti-LC3B antibody (1:200) after the cell membranes had been broken in 0.5% Triton-X at 4°C overnight. The secondary antibody was Alexa Fluor 647-conjugated goat-anti-rabbit IgG (H+L) secondary antibody (1:200). After incubating with secondary antibody at 37°C in the dark, all sections were finally stained with 4′,6−diamino−2−phenylindole (DAPI, 1:10000) to distinguish cell nuclei. Immunofluorescence images were collected using a light microscope (Eclipse 80i, Nikon, Japan).

### Immunohistochemical Staining

Immunohistochemical staining for RMCs (TNFα, IL-1β, IL-6, and LC3B) was performed with 4-μm paraffin sections. First, all sections were subjected to deparaffinage in xylene followed by graded ethanol before being microwaved on medium for 10 min in 10 mM trisodium citrate (pH 6) to retrieve intracellular antigens. Sections were then incubated for 10 min with 0.6% hydrogen peroxide. Next, 5% sheep serum was used to block nonspecific detection. Sections were then incubated with corresponding primary antibody (LC3B, 1:200) in 5% bovine serum albumin (BSA) at 4°C overnight. After washing in PBS for 3 min thrice, sections were incubated with biotinylated goat antibodies (antirabbit IgG, 1:200, San Francisco, CA, USA) or biotinylated rabbit antibody (antigoat IgG, 1:200, Zymed, San Francisco, CA, USA) for 1 hour followed by being placed in ABC solution (ABC Kit) (Vector Laboratories) for 1 hour at room temperature and then developed with 3,3-diaminobenzidine (DAB) (Sigma Chemical Co.) to produce a brown color. Images were then collected using a light microscope (Eclipse 80i, Nikon, Japan).

### Western Blotting Analysis

Cells were seeded in 10-cm dishes and treated with indicated doses of extract and constituents for the indicated time. Cells of each were then respectively collected with IP lysis buffer and shaken at 4°C. Next, all of the liquid samples were boiled with a quarter volume of 10X loading buffer (0.05 g bromophenol blue, 50 mM Tris-HCl (pH 6.8), 10% glycerol, 2% SDS, and 10 mL β-mercaptoethanol) for 10 min. Equal amounts of protein were loaded in each lane of SDS-PAGE gels and electrophoretically blotted onto PVDF membranes. The PVDF membrane was then blocked by using 5% skim milk at room temperature for one hour and incubated with corresponding antibodies at 4°C overnight. After rinsing with TBS-T for 5 min thrice, all membranes were incubated with mouse or rat horseradish peroxidase (HRP)-conjugated secondary antibody at indoor temperature for one hour. The protein bands were detected using ECL reagents and visualized by a gel imaging system (Bio-Rad, USA, 721BR08110).

### Real-Time PCR

Total RNA was extracted from RMCs using the RNAsimple Total RNA Kit and was reverse transcribed according to the manufacturer’s protocol. Amplification reactions were performed on real-time fluorescent quantitative PCR. The primer set for RT-PCR is shown in **Table 1**. All of the primers were synthesized by Sangon (Shanghai, China).

**Table 1:** The primer sequences for Real-Time PCR.

GenePrimer sequence (5′→3′)		
TNFα	**F:**CATCTTCTCAAAATTCGAGTGACAA	**R:**TGGGAGTAGACAAGGTACAACCC
IL-6	**F:**AAAGAGTTGTGCAATGGCAATTCT	**R:**AAGTGCATCATCGTTGTTCATACA
IL-1β	**F:** TGCCACCTTTTGACAGTGATG	**R:**AAGGTCCACGGGAAAGACAC
Beclin1	**F:**CCAGAGAAGAATGCTGTACGAAT	**R:**CCAGTTGGTAACAATGCCATGT
PINK1	**F:**TTCTTCCGCCAGTCGGTAG	**R:**CTGCTTCTCCTCGATCAGCC
Parkin	**F:**TCTTCCAGTGTAACCACCGTC	**R:**GGCAGGGAGTAGCCAAGTT
LC3B	**F:**TTATAGAGCGATACAAGGGGGAG	**R:**CGCCGTCTGATTATCTTGATGAG
β-actin	**F:**GGCTGTATTCCCCTCCATCG	**R**:CCAGTTGGTAACAATGCCATGT

### High Performance Liquid Chromatography Analysis of APF

Calycosin, β-ecdysterone, ferulic acid, Astragaloside I, and Astragaloside IV, the five main effective constituents of APF, were assayed with an AgilentHigh Performance Liquid Chromatography (HPLC) system equipped with LC solution software and a UV spectrophotometer. An octadecylsilane-bonded rubber column (C18, 4.6 mm × 250 mm, 5 μm) was used and kept at room temperature. The UV spectrophotometer was set at 350 nm. The percentage composition of the mobile phase varies due to the properties of different samples, but they were all gradient eluted at a flow rate of 1 ml/min. The samples specially used for experiments were extracted according to the processing method provided in the Chinese pharmacopeia (2015 edition); 10 μL of each blank control solution and gradient concentrations of the sample solution were added to brown sample bottles. The details of the mobile phase and its variation in ratio with time are shown in **Table 2**.

**Table 2:** Mobile phase condition of chromatographic separation.

Target	Time (min)	**Mobile Phase Ratio**
Calycosin	0~20	Acetonitrile : 0.2% formic acid solution = 20→40 : 80→60
	20~30	Acetonitrile :0.2% formic acid solution = 40 : 60
β-ecdysterone	0~30	Acetonitrile : H_2_O-0.2% formic acid solution = 16 : 84 : 0.1
Ferulic acid	0~30	Acetonitrile :0.085% phosphoric acid solution = 17:83
Astragaloside I	0~60	Acetonitrile :H_2_O = 32 : 68
Astragaloside IV	0~60	Acetonitrile : H_2_O = 32 : 68

### Statistical Analysis

One-way ANOVA and independent-samples t-test were applied to analyzing the groups of samples in SPSS 21.0 software (Inc., Chicago, IL, USA). GraphPad Prism 7 software was applied for image production and output. *P-*values of less than 0.05 were considered statistically significant.

## Results

### Analysis of the Main Components of APF

The main component profile of APF was analyzed *via* HPLC–UV. The representative chromatogram is shown in **Supplementary Figure 1**. The identification of APF constituents was based on the retention time and the UV spectrum in comparison with authentic standards at a wavelength of 350 nm. The main components of APF are Astragaloside I, Astragaloside IV, Ferulic Acid, Calycosin, and β-ecdysterone.

### Effects of APF on the General Condition of DN Mice

**Figure 1:**
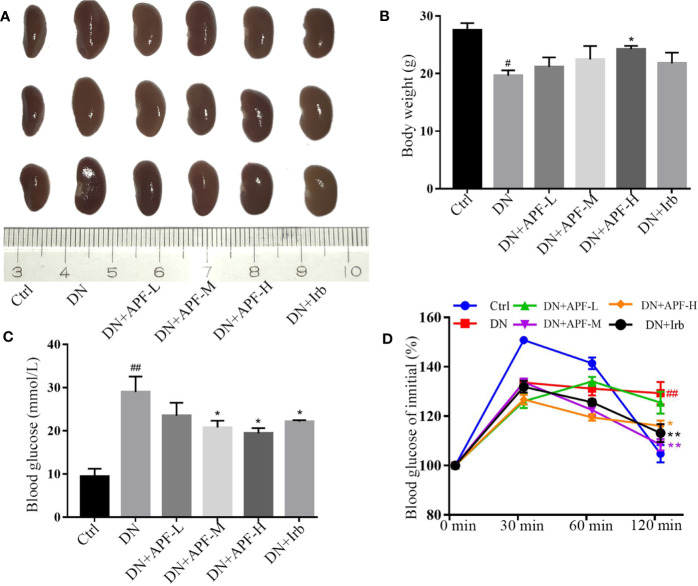
Effects of APF on the general condition of DN mice. **(A, B)** APF administration effectively reduced renal volume expansion and rescued body weight loss. **(C, D)** APF treated hyperglycemia and improve glucose tolerance in DN mice. Data are expressed as the mean ± SEM. ^#^P < 0.05, ^##^P < 0.01 versus the Ctrl group; *P < 0.05, **P < 0.01 versus the DN group

**Figure 2:**
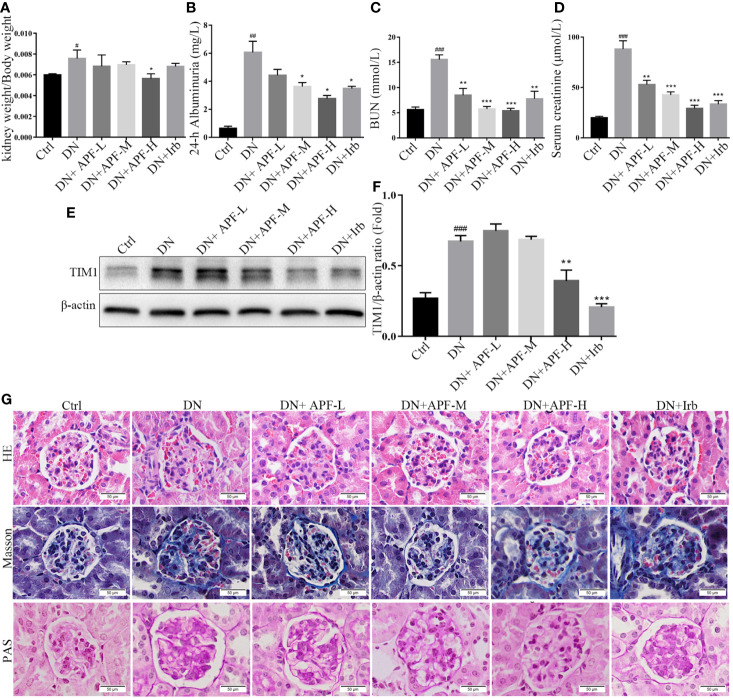
Effects of APF on renal injuries in DN kidneys. **(A–D)** Treatment of APF reduced kidney weight/body weight (ratio), 24-h albuminuria, BUN, and Scr of DN mice. **(E, F)** Effect of APF on protein level of TIM1 was detected by Western blotting analysis. **(G)** HE staining, Masson staining, and PAS staining of mouse kidneys. (Scale bar = 50 µm). ^#^*P* < 0.05, ^##^*P* < 0.01, ^###^*P* < 0.001 versus the Ctrl group; ^*^P < 0.05, ^**^P < 0.01, ^***^P < 0.001 versus the DN group.

**Figure 3:**
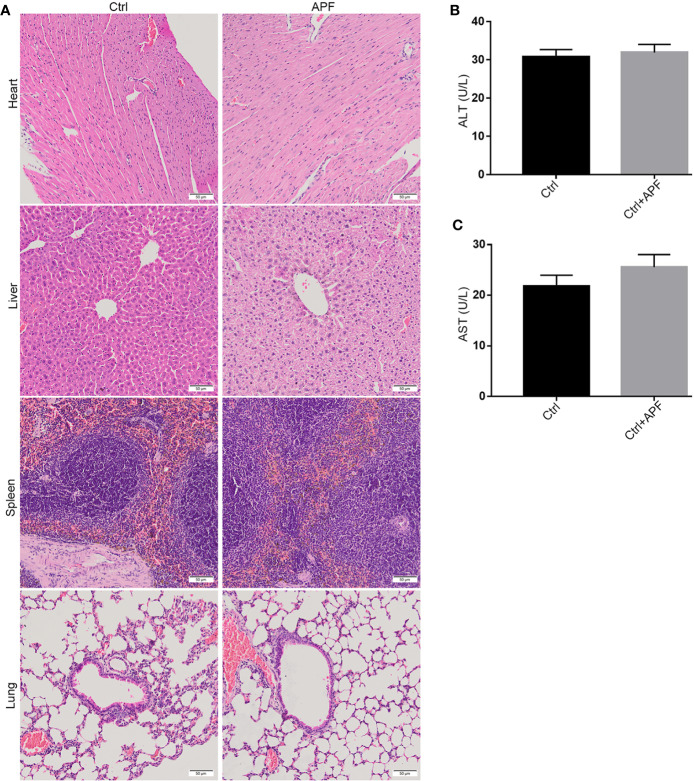
Effects of APF on major organs except for kidneys. **(A)** The HE staining demonstrated that no pathologic change can be observed in heart, liver, spleen, and lung after APF administration in mice. **(B, C)** APF administration did not upregulate the ALT and AST levels in serum of APF group compared to the Ctrl group. (Scale bar = 50 µm).

Based on the findings of other researchers and the clinical features of DN patients, the DN mice did indeed suffer from DN, as they exhibited enlargement of kidneys (**Figure 1A**) and significant weight loss (**Figure 1B**). The treatment with APF observably rescued the body weight and reduces the kidney weight/body weight in DN mice. However, fasting blood glucose, one of the features of DN, was only mildly reduced after APF treatment (**Figure 1C**), as was glucose tolerance (**Figure 1D**). Scr and serum BUN can clearly reflect the status of the kidney, as is recognized in both clinical and scientific research, and 24-h albuminuria can reflect the degree of glomerular dysfunction. Compared with control mice, 24-h albuminuria, BUN, and Scr significantly increased in DN mice and then remarkably decreased in those treated with APF, as expected (**Figures 2B–D**). After intragastric administration of APF in the normal group, there were no significant pathologic changes to heart, liver, spleen, and lung compared to mice without APF intervention (**Figure 3A**), and the measurements of alanine aminotransferase (ALT) and aspartate aminotransferase (AST), the two most intuitive markers of liver function, in the serum of mice showed that APF did not place a significant functional burden on the liver (**Figures 3B, C**).

### APF Reduced Histological Damage and Mesangial Matrix Deposition in DN Kidneys

In previous studies on DN in humans and experimental animals, mesangial matrix hyperplasia, basement membrane thickening, and the deposition of renal tubulointerstitial fibrosis in the kidney were considered typical pathological features of DN. In this research, histological analysis by HE staining and PAS staining revealed the occurrence of mesangial matrix expansion, extracellular matrix deposition, and renal tubulointerstitial fiber deposition in the kidneys of DN mice (**Figure 2G**). The treatment with APF visibly ameliorated these histological renal pathological hyperplasia phenotypes in DN mice (**Figure 2G**).

### APF Ameliorated DN-Induced Inflammation, Intrarenal Injury, and Fibrosis in DN Kidneys

**Figure 4:**
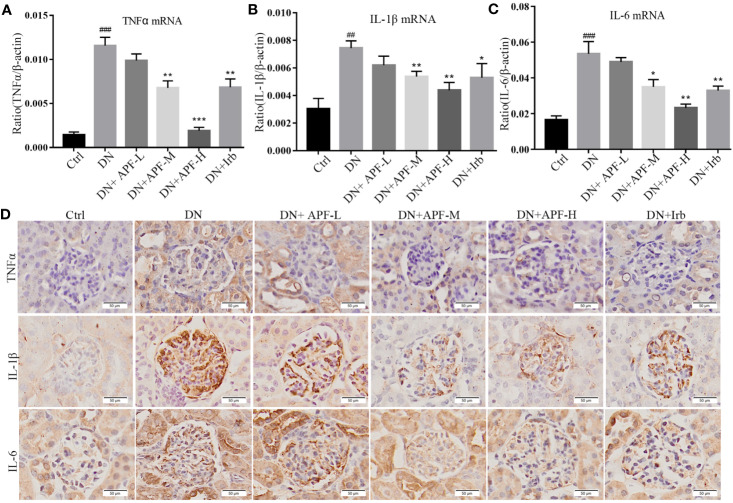
Effects of APF on inflammatory markers in DN kidneys. **(A–C)** The mRNA expressions of TNFα, IL-1β and IL-6 were measured by real-time PCR analysis. **(D)** Immunohistochemical staining of TNFα, IL-1β, and IL-6 in mouse kidneys. (Scale bar = 50 µm). ^##^*P* < 0.05, ^###^*P* < 0.001 versus the Ctrl group; ^*^P < 0.05, ^**^P < 0.01, ^***^P < 0.001 versus the DN group.

Inflammation is also a leading pathological feature of DN, so we examined the expression of inflammatory factors in kidney of DN mice through real-time PCR and immunohistochemistry. IHC staining revealed that TNFα, IL-1β, and IL-6 significantly increased in DN kidneys, while treatment with APF remarkably reduced its secretion in the kidneys of these treated mice (**Figure 4D**). The quantitative analysis of real-time PCR also more clearly revealed the same variation tendency of TNFα, IL-1β, and IL-6 in the renal cortical tissues among the control group, DN group, and intervention groups (**Figures 4A–C**). Immunoblotting assay for proteins extracted from renal cortex and boiled in SDS-loading buffer revealed that the protein level of TIM1 could also be reduced by APF treatment (**Figures 2E, F**).

### APF Upregulated Autophagy, Suppressed mTOR, and Activated the PINK1/Parkin Signaling Pathway in DN Kidneys

**Figure 5:**
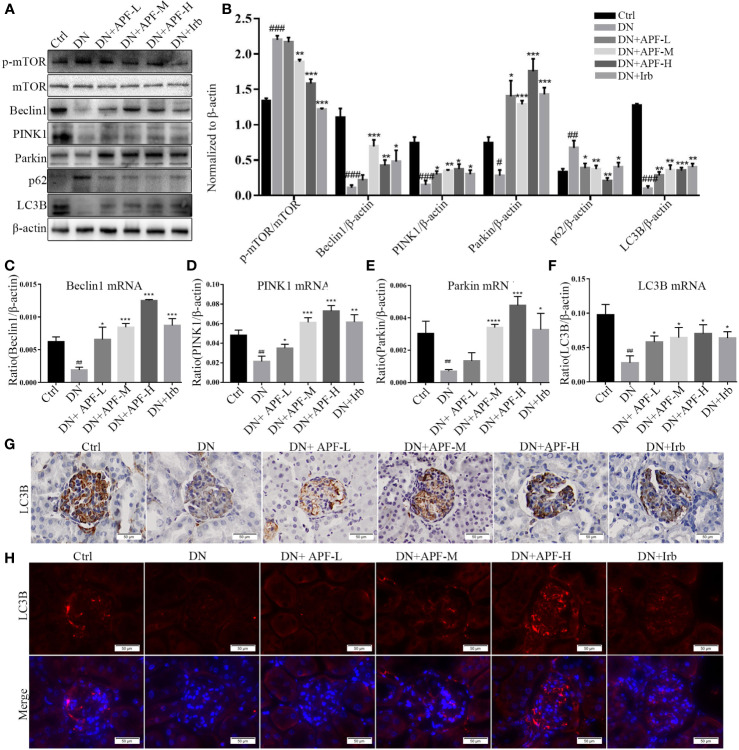
Effects of APF on autophagy markers in DN kidneys through the mTOR/PINK1/Parkin pathway. **(A, B)** Effect of APF on protein levels of p-mTOR, mTOR, Beclin1, PINK1, Parkin, and LC3B, detected by Western blotting analysis. **(C–F)** Effect of APF on mRNA expression of Beclin1, PINK1, Parkin, and LC3B according to real-time PCR analysis. **(G)** Immunohistochemical staining of LC3B in DN kidneys. **(H)** Immunofluorescence staining of LC3B in DN kidneys. (Scale bar = 50 µm). ^#^*P* < 0.05, ^##^*P* < 0.01, ^###^*P* < 0.001 versus the Ctrl group; ^*^P < 0.05, ^**^P < 0.01, ^***^P < 0.001, ****P < 0.0001 versus the DN group.

Autophagy involving macroautophagy and mitochondria is considered to be an effective method of physiological degrading damaged organelles and mitochondria, which plays an important role in slowing down the progression of DN. We therefore examined changes in the protein and gene levels of molecules involved in the autophagy pathway through immunoblotting analysis, real-time PCR quantitative analysis, immunofluorescence, and immunohistochemical staining. The protein molecule mTOR unquestionably plays important roles in regulating mitophagy. To determine the activation of mTOR, we analyzed the protein expression of p-mTOR and mTOR in different groups of mice. The immunoblotting analysis showed that the activation of p-mTOR significantly decreased in comparison to total mTOR in DN kidneys and that the activity of the mTOR molecule decreased after APF intervention (**Figures 5A, B**). Additionally, the downstream protein molecules Beclin1, PINK1, and Parkin obviously decreased in DN kidneys and was largely rescued by APF intervention (**Figures 5A, B**). The mRNA levels of Beclin1, PINK1, Parkin, and LC3B varied similarly to their protein levels in the different groups (**Figures 5C–F**). Immunohistochemical staining revealed a distinct reduction of LC3B expression in DN kidneys (**Figure 5G**), and immunofluorescence staining also revealed that APF treatment significantly restored LC3B expression, which had been reduced in DN kidneys (**Figure 5H**). p62, which functions by combining with LC3B, exhibited an opposite tendency to LC3B in protein expression, indicating that autophagosome decreased in DN kidneys but rose after APF intervention (**Figures 5A, B**).

### APF Protected HG-Induced RMCs From Injury and Autophagy Dysfunction

**Figure 6:**
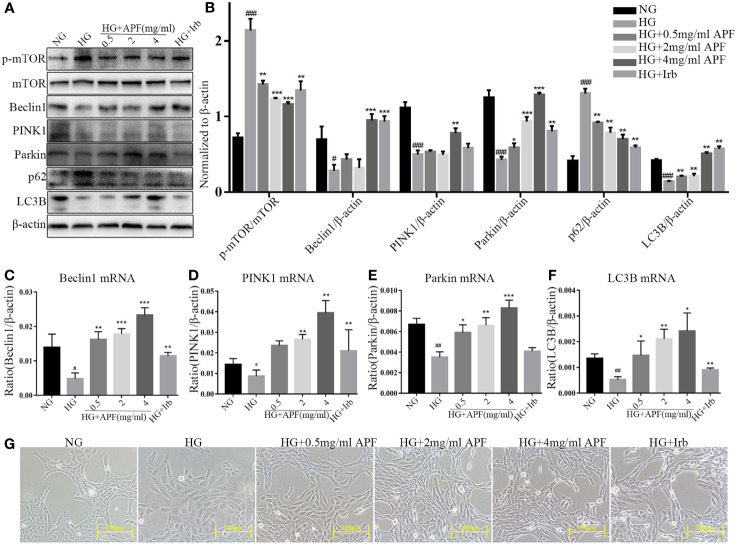
Effects of APF-containing serum on autophagy markers in RMCs under HG-induced injury and dysfunction. **(A, B)**. Effect of APF-containing serum on protein levels of p-mTOR, mTOR, Beclin1, PINK1, Parkin, and LC3B, detected by Western blotting analysis. **(C–F)** Effect of APF-containing serum on mRNA expression of Beclin1, PINK1, Parkin, and LC3B according to real-time PCR analysis. **(G)** RMCs were examined under an optical microscope. (Scale bar = 100 µm). ^#^*P* < 0.05, ^##^*P* < 0.01, ^###^*P* < 0.001 versus the Ctrl group; ^*^P < 0.05, ^**^P < 0.01, ^***^P < 0.001 versus the HG group.

To determine the most suitable D-(+)-glucose dosage as the HG condition, RMCs were cultured respectively in 5 mM, 25 mM, and 30 mM glucose medium. Mannitol at 25 mM and 30 mM was used as blank control to eliminate the influence of increased osmotic pressure from respective concentration of glucose. In order to simulate the DN internal environment, 30 mM glucose for 48 hours was selected to induce an autophagy-deficiency model in RMCs (**Supplementary Figures 2A, B**). In order to determine the upper limit for APF-containing serum, the cytotoxicity was assessed by CCK-8 assay, and a cytotoxic effect occurred when the dosage was over 4 mg/ml (**Supplementary Figure 2C**). To study the effect of APF-containing serum on mitophagy in renal injury, RMCs were cultured at multiple concentrations ranging from 0.1 to 6 mg/ml. Exposure to APF-containing serum at concentrations ranging from 0.1 to 4 mg/mL did not produce any significant effect on the 24-h survival rate of RMCs. Based on these results, APF concentrations < 4 mg/mL were applied in the subsequent experiments (**Supplementary Figures 2D, E**). Increasing the concentration of APT in serum dissolved in basal culture medium can enhance its blocking effect on mesangial expansion, as shown in photographs obtained under a light microscope (**Figure 6G**). We next investigated the mechanisms whereby APF recovered HG-stimulated RMCs. Compared with the HG group, the mRNA expressions of Beclin1, PINK1, and Parkin were significantly upregulated in RMCs exposed to gradient concentrations of APF for 24 hours (**Figures 6A, B**), and a similar degree of variation was observed in HG-induced RMCs as in *in vivo* experiments. The mRNA expression of autophagy-related protein markers was upregulated in RMCs after APF-containing serum intervention (**Figures 6C–F**).

### APF Activates the mTOR/PINK1/Parkin Pathway and Ameliorates Cellular Injury and Autophagy Dysfunction in HG-Stimulated RMCs

**Figure 7:**
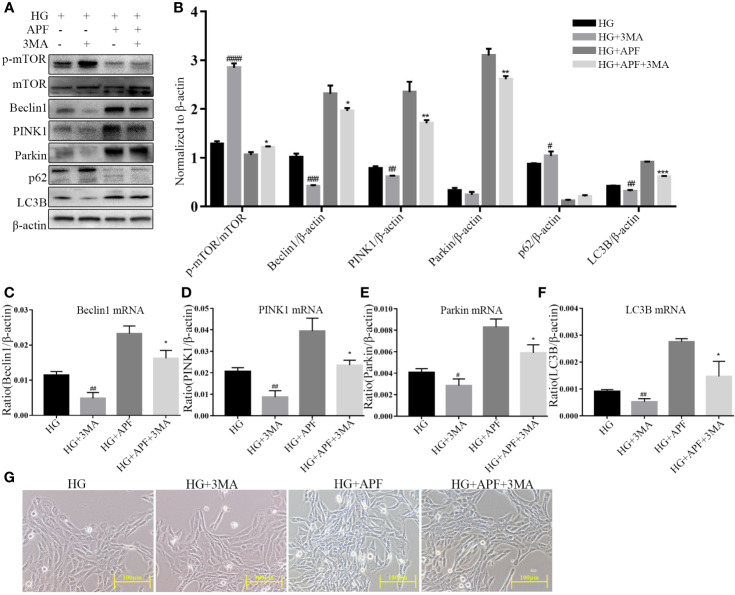
Effects of APF on autophagy markers and PI3K inhibitor 3-MA under HG condition. **(A, B)** Effect of APF-containing serum and 3-MA (100 μM) on protein levels of p-mTOR, mTOR, Beclin1, PINK1, Parkin, and LC3B under HG stimulation, detected by Western blotting analysis. **(C–F)** Effects of APF-containing serum and 3-MA (100 ìM) on mRNA expression of Beclin1, PINK1, Parkin, and LC3B according to real-time PCR analysis. **(G)** RMCs were observed under an optical microscope. (Scale bar = 100 µm). ^#^*P* < 0.05, ^##^*P* < 0.01, ^###^*P* < 0.001, ^####^*P* < 0.001 versus the HG group; ^*^P < 0.05, ^**^P < 0.01, ^***^P < 0.001 versus the HG+APF group.

In order to further detect how APF-containing serum works in RMCs under the HG condition, we inhibited the activity of autophagy with its antagonist 3-MA (100 µM). RMCs under HG stimulation became even bigger and rounder after 3-MA intervention (**Figure 7G**). 3-MA is a selective PI3K inhibitor, and significant upregulation of p-mTOR was observed in immunoblotting analyzes in HG-stimulated RMCs after 3-MA intervention, as expected (**Figures 7A, B**). We also found that 3-MA intervention significantly reduced the expressions of Beclin1, PINK1, Parkin, and LC3B (**Figures 7A, B**). As expected, gene detection with real-time PCR showed that 3-MA could also reduce Beclin1, PINK1, Parkin, and LC3B, whether there was HG stimulation or not (**Figures 7C–F**). As previously mentioned, APF-containing serum can indeed ameliorate the change in size and shape of RMCs and rescue the level of autophagy and activate the PINK1/Parkin signaling pathway in them. These results suggested that the autophagy inhibition by 3-MA weakens the therapeutic effect of APF-containing serum and its function of suppressing mTOR and activating the PINK1/Parkin signaling pathway. Above all, blocking the autophagy activity by its antagonist 3-MA muted APF’s protection against HG-induced RMC injury, suggesting that APF works against DN *via* promoting the autophagy level meditated by the mTOR/PINK1/Parkin signaling pathway in RMCs.

## Discussion

In previous studies, APF has been widely used in the clinical treatment of CKD (Wen et al., 2014). The five components of APF have been proven to have excellent anti-inflammatory, anti-fibrosis, anti-oxidative stress, and autophagy restoration effects and have been widely used in the treatment of DN in China. As the main source of the medicinal effects of *Astragalus mongholicus* Bunge, Astragaloside IV has been shown to significantly attenuate pro-inflammatory cytokines such as TNFα and IL-1β in UUO mice and LPS-induced renal epithelial cells by regulating the TLR4/NF-кB signaling pathway (Zhou et al., 2017) and to significantly improve the condition of abnormal kidney injury by regulating PINK1/Parkin-mediated mitophagy in db/db mice (Liu et al., 2017). Not only Astragaloside IV but also other extracts from *Astragalus mongholicus* Bunge, such as flavonoids and polysaccharides, have significant protective effects on tissue damage through antioxidant mechanisms (Shahzad et al., 2016). Calyosin, which is one of the main components of *Astragalus mongholicus* Bunge, can significantly improve STZ-induced renal injury and dysfunction by regulating the IL-33/ST2 signaling pathway, inflammatory cytokines, oxidative stress, and fibrosis (Elsherbiny et al., 2019). Among the many standard ingredients that we successfully tested, Ferulic acid is one of the main components of Angelica sinensis (Oliv.) Diels, and its pharmacological free-radical scavenging, anti-inflammatory, anti-oxidation, antioxidant enzyme activity increasing, and lipid peroxidation alleviation effects have been shown to play an important role in the recovery of CKD (Nankar et al., 2017). β-ecdysterone is mainly responsible for the pharmacological effect of *Achyranthes bidentata* Blume and can not only lower the blood glucose of DM patients but also normalize the levels of stable NO metabolites to protect the kidneys from oxidation and nitrosation stress (Korkach et al., 2007). Although these monomer Chinese medicines all play their respective functions in the treatment of DN, according to the practical and traditional Chinese medicine theory, it is rare to apply a single monomer Chinese medicine to treat DN. In TCM theory, “JUN,” “CHEN,” and “SHI” work together. Among them, “JUN” medicines act as the most important component aimed at the disease. They may not necessarily be the components in the highest quantities in the formula, but their quality matters more, as they are the main source of pharmacological action. “CHEN” medicines work to help strengthen the effects of “JUN” medicines on diseases. “SHI” actually means “guidance,” and the function of such components is to guide the drugs within the formula to the lesion, enabling better targeting. Thus, most of them are specialized in clearing turbidity and enhancing metabolism. As such, TCM theory has always believed that the superposition of the functions of different traditional Chinese medicines, that is to say, the reasonable compatibility of traditional Chinese medicines, can play a more important role in practical clinical application. Based on TCM theory and the results of this study, we can speculate that APF is a potential therapeutic option in the treatment of CKD *via* regulating autophagy.

With the development of society, change in people’s dietary habits, and acceleration of the pace of life, the incidence of DM is also increasing rapidly. DM and its further complications have become an important killer, threatening human health worldwide, especially DN. It has been mentioned before that genetic and environmental factors have already been proved to be related to the occurrence and development of DN, including genetic susceptibility, sedentary lifestyle, hypertension, persistent hyperglycemia, and dyslipidemia. Moreover, it has been confirmed in epidemiological investigations that maintaining a healthy weight, having a healthy diet, maintaining physical activity, quitting smoking, and responsible drinking will help slow the course of DM and then reduce the incidence of complications such as DN (Zheng et al., 2018). In recent years, many studies have found that an increase in mTOR activity and a subsequent decrease of autophagy-related molecular activities such as those of PINK1/Parkin and LC3B can be observed in kidneys of DN animal models (Gödel et al., 2011; Xiao et al., 2017; Li et al., 2017), which may be one of the mechanisms leading to kidney damage. Among them, strict inhibition of mTOR activity is essential for maintaining glomerular podocyte function and thereby reducing proteinuria and glomerulosclerosis (Gödel et al., 2011). This process involves a variety of mechanisms; autophagy is just one part of it.

Although the inflammatory immune response associated with CKD is very different from that in acute kidney injury caused by ischemia or infection, it is still undeniable that it plays a vital role in the pathophysiology of DN (Tesch, 2017; Moreno et al., 2018). In this research, a significant increase in the expression of inflammatory factors like TNFα, IL-1β, and IL-6 could indeed be detected in DN model mice induced by STZ combined with a high-fat and high-sugar diet for nearly three months. Autophagy and its degradation through the lysosomal pathway play an important role in eliminating damaged organelles (including damaged mitochondria) and maintaining the homeostasis of the body. This process is mediated by evolutionarily conserved autophagy-related genes (ATGs). Numerous pieces of evidence indicated that mitochondrial dysfunction might be one of the pathomechanisms of DN (Forbes et al., 2008; Bock et al., 2013; Coughlan and Sharma, 2016), so it might be a considerable problem if damaged organelles or mitochondria cannot be removed by autophagy. In our study, the immunoblotting and IHC results of relevant receptors and molecules in the DN kidneys were consistent with those of previous studies, which showed a decrease in Beclin1 and LC3B expression, indicating that mTOR and PINK1/Parkin signaling pathway-mediated autophagy deficiency indeed existed in DN mouse kidneys. This raises the question of how autophagy deficiency affects the inflammation and damage of DN kidneys. Previous research revealed that autophagy deficiency severely limited the clearance of advanced glycosylated end products (AGEs), thus aggravating the accumulation of damaged mitochondria and organelles (Yamamoto-Nonaka et al., 2016). Furthermore, autophagy deficiency further increased hypoxia and endoplasmic reticulum stress, thereby increasing the susceptibility of renal tubular cells to damage (Jeon et al., 2015). Simultaneously, inhibition of autophagy also leads to the deposition of collagen in interstitium, which is caused by deficiency of the degradation function (Li et al., 2016). Mammalian target of rapamycin (mTOR), a Ser/Thr protein kinase of rapamycin-targeted protein, could be activated by AKT and MAPK signaling to inhibit autophagy and down-regulated by AMPK and p53 signaling to promote autophagy (Agarwal et al., 2015). A lot of research has shown that mTOR activity is significantly increased in CKDs in both humans and mice (Zhang et al., 2014); a similar phenomenon was observed in our research. Induction through HG is a well-recognized model for simulating the HG environment in DM, and mTOR activity increased in HG-induced RMCs, just as in previous studies (Zhan et al., 2018), simulating the HG environment in DM of humans and animals. To sum up, activation of mTOR suppressed the level of macroautophagy, and thereby aggravated inflammation and damage, in DN kidneys. Besides macroautophagy, the level of PINK1/Parkin signaling pathway-meditated mitophagy in kidneys of STZ-induced DN mice and DN patients diagnosed by biopsy were significantly reduced, with severe podocyte damage and proteinuria (Zhou et al., 2019). Previous studies have shown that mitochondrial dysfunction is the main source of reactive oxygen species and mediates the inflammation activation by regulating IL-1β (Lin et al., 2016). Nevertheless, in our experiment, autophagy antagonist 3-MA was used to inhibit the level of autophagy in RMCs and could significantly block the protection afforded by APF-containing serum against HG-evoked cell injury. Therefore, we speculate that promoting mTOR-mediated macroautophagy and PINK1/Parkin signaling-mediated mitophagy is a therapeutic mechanism that reduces renal inflammation, fibrosis, and injury in DN kidneys.

## Conclusion

In summary, these experimental results showed that APF could rescue the impairment of renal structure and kidney function and reduce the inflammatory level in DN kidneys. On the other hand, APF could also enhance mTOR/PINK1/Parkin signaling both *in vivo* and *in vitro*. In conclusion, these findings provide novel insight into the remarkable anti-inflammatory effect and anti-fibrotic effect of APF, which are related to its autophagy redemption effect.

## Data Availability Statement

All datasets generated for this study are included in the article/**Supplementary Material**.

## Ethics Statement

The animal study was reviewed and approved by Ethics committee of Southwest Medical University.

## Author Contributions

LW, R-ZT, DW: funding acquisition, investigation, and writing—original draft preparation. C-YZ, J-CL, XZ, HD, XL: methodology and validation. J-MF: supervision. LW, D-YD, X-SX: project administration and writing—review and editing. All authors contributed to the article and approved the submitted version.

## Funding

This study is supported by a Luzhou-Southwest Medical University Joint Project (2016LZXNYD-T05) and Joint Platform Projects (2017LZXNYD-P01, 2018LZXNYD-PT03), the Southwest Medical University and Affiliated Traditional Medicine Hospital Joint program (2018XYLH-032), the Luzhou Science and Technology Project (2016-S-68(4/8) and 2011-108), the Luzhou Municipal-Southwest Medical University Joint Special Grant for the Introduction of High-Level Talents (Chen Chen Team and Lan Hui-Yao Team), the Sichuan Traditional Chinese Medicine Administration Project (2018JC037), and the Health Commission of Sichuan Province Project (18PJ367).

## Conflict of Interest

The authors declare that the research was conducted in the absence of any commercial or financial relationships that could be construed as a potential conflict of interest.

## Supplementary Material

Supplementary Figure 1 Analysis of the main medicinal components of APF. High-performance liquid chromatography detected the effective components in APF: Astragaloside I, Astragaloside IV, Ferulic Acid, Calycosin, and β-ecdysterone.

Supplementary Figure 2 Preparation of appropriate doses of Glucose and APF-containing serum in RMCs. (A-B) In order to establish an HG-induced RMC model, 5 mM, 25 mM, and 30 mM glucose were administrated at 24 hours and 48 hours, respectively. The same concentrations of mannitol were prepared to eliminate the influence of osmotic pressure. The expression of Parkin in each group was examined by Western blotting, and we observed that mannitol had no effect on Parkin expression at 48 hours compared with the NC group. Moreover, a 30-mM glucose concentration acting on RMCs for 48 h had a significant inhibitory effect on Parkin, so 30 mM glucose treatment of RMCs for 48 h was selected for the subsequent experiments. (C-E) We freeze-dried powder of APF-containing serum at low temperature and negative pressure and dissolved it in the basic medium. According to the results of CCK-8 assays and the detection of autophagy-related indexes by Western blotting, we finally determined that 0.5 mg/ml, 2 mg/ml, and 4 mg/ml were appropriate final concentrations of APF-containing serum for the subsequent cell experiments. ^#^*P* < 0.05, ^##^*P* < 0.01, ^###^*P* < 0.001 versus the 5 mM group; ^**^*P* < 0.01, ^***^*P* < 0.001 versus the HG group.

## Abbreviations

DM, diabetes mellitus; DN, diabetic nephropathy; ESRD, end-stage renal disease; STZ, streptozocin; APF, *astragalus mongholicus* Bunge and Panax notoginseng (Burkill) F.H. Chen formula; DAPI, 4′,6-diamidino-2-phenylindole; PAS staining, periodic acid-Schiff staining; HE staining, hematoxylin-eosin staining; Scr, serum creatinine; BUN, blood urea nitrogen; PINK1, PTEN-induced putative kinase 1; 3-MA, 3-Methyladenine; HO-1, heme oxygenase-1; ROS, reactive oxygen species; AKI, acute kidney injury; HG, high glucose; NS, normal saline; RMC, renal mesangial cell; FBG, fasting blood glucose; TNFα, tumor necrosis factor alpha; BSA, bovine serum albumin; DAB, 3,3-diaminobenzidine; HPLC, high-performance liquid chromatography; IL-1β, Interleukin-1 beta; IL-6, Interleukin-6; TIM1(KIM1), kidney injury marker 1; ALT, alanine aminotransferase; AST, Aspartate aminotransferase; CKD, chronic kidney disease; AGEs, advanced glycosylated end products.
