# Supplementary figures and images for: Astragalus mongholicus Bunge and Panax notoginseng (Burkill) F.H. Chen Formula for Renal Injury in Diabetic Nephropathy—In Vivo and In Vitro Evidence for Autophagy Regulation
Source: Front Pharmacol. 2020 Jun 12;11:732. doi: 10.3389/fphar.2020.00732 (PMC7303297; doi:10.3389/fphar.2020.00732)

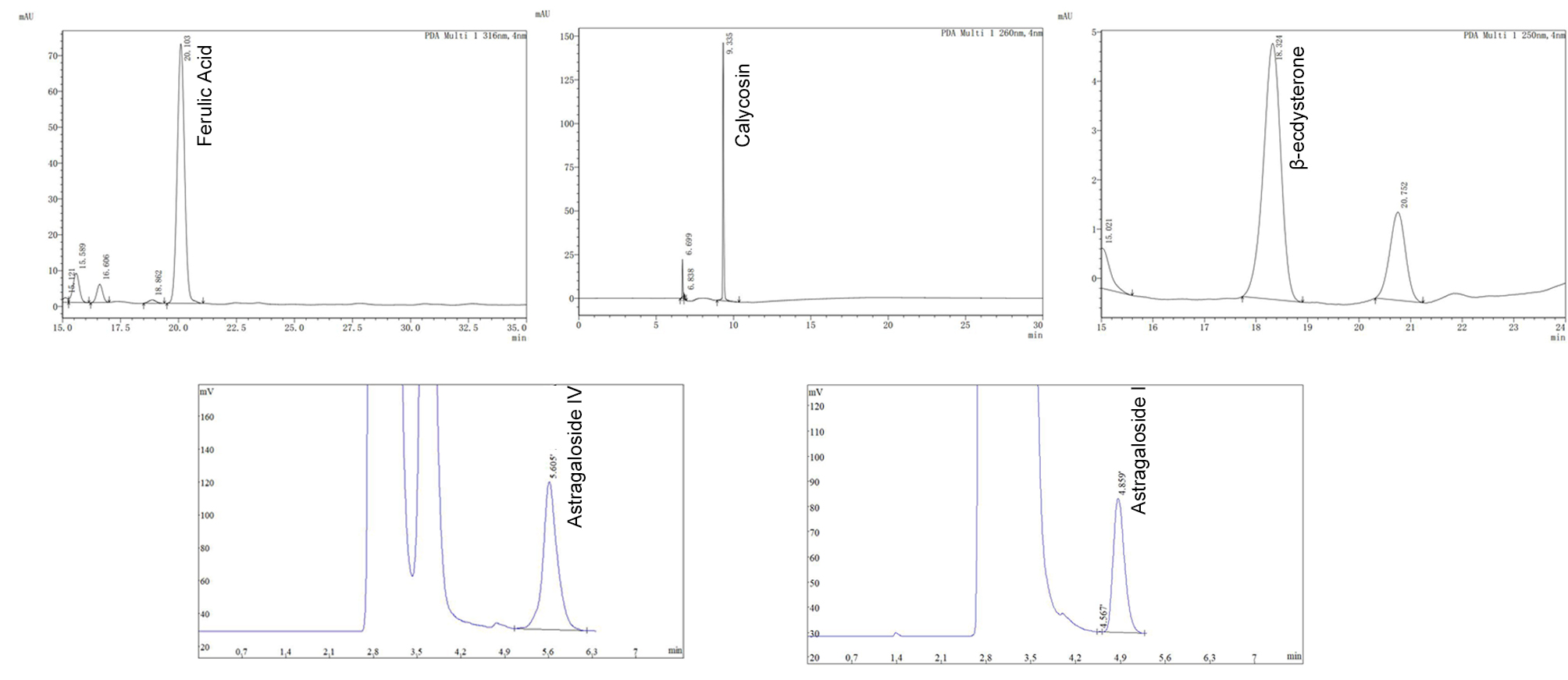

Supplement: Supplementary Figure 1 — Analysis of the main medicinal components of APF. High-performance liquid chromatography detected the effective components in APF: Astragaloside I, Astragaloside IV, Ferulic Acid, Calycosin, and β-ecdysterone. [file Image_1.jpeg]

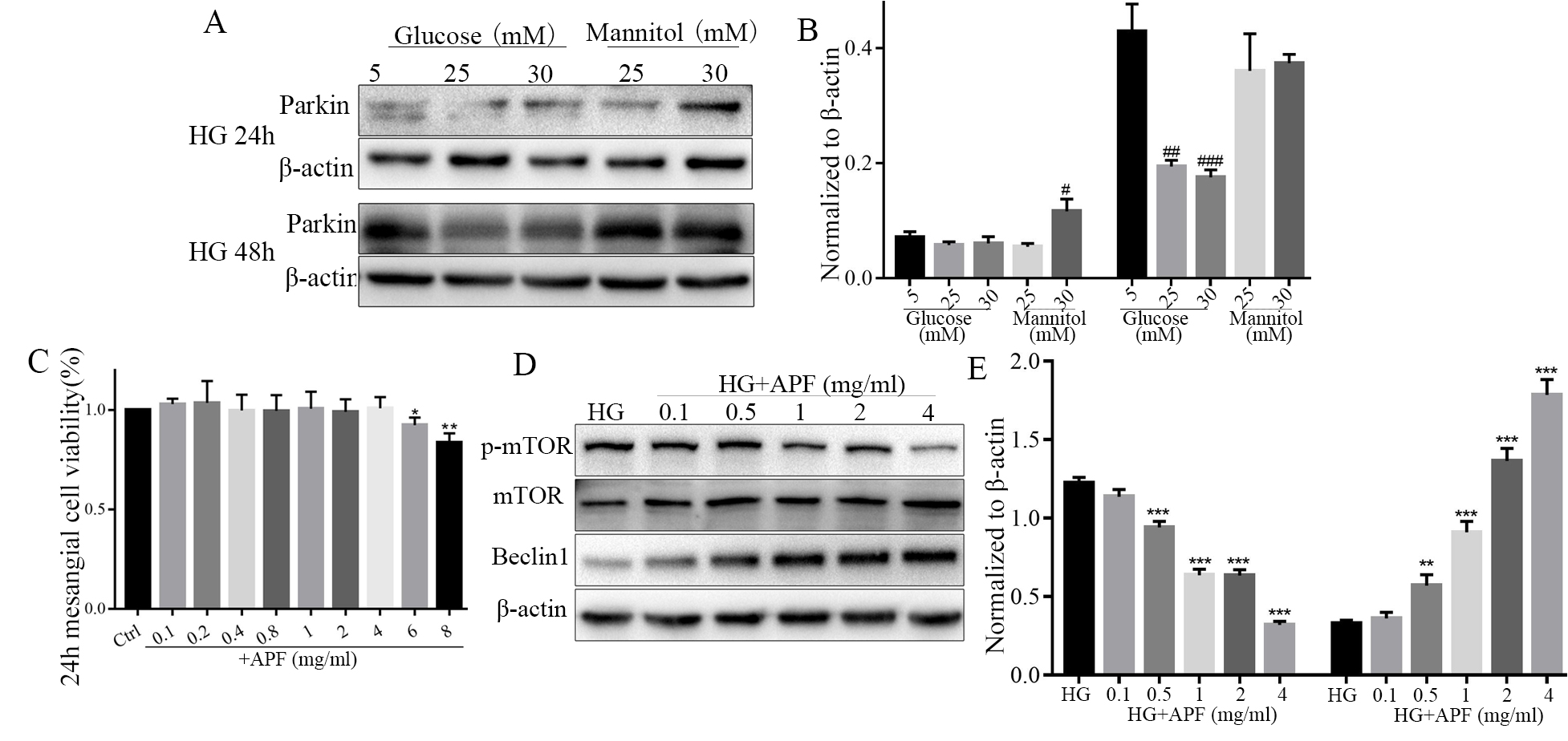

Supplement: Supplementary Figure 2 — Preparation of appropriate doses of Glucose and APF-containing serum in RMCs. (A-B) In order to establish an HG-induced RMC model, 5 mM, 25 mM, and 30 mM glucose were administrated at 24 hours and 48 hours, respectively. The same concentrations of mannitol were prepared to eliminate the influence of osmotic pressure. The expression of Parkin in each group was examined by Western blotting, and we observed that mannitol had no effect on Parkin expression at 48 hours compared with the NC group. Moreover, a 30-mM glucose concentration acting on RMCs for 48 h had a significant inhibitory effect on Parkin, so 30 mM glucose treatment of RMCs for 48 h was selected for the subsequent experiments. (C-E) We freeze-dried powder of APF-containing serum at low temperature and negative pressure and dissolved it in the basic medium. According to the results of CCK-8 assays and the detection of autophagy-related indexes by Western blotting, we finally determined that 0.5 mg/ml, 2 mg/ml, and 4 mg/ml were appropriate final concentrations of APF-containing serum for the subsequent cell experiments. #P < 0.05, ##P < 0.01, ###P < 0.001 versus the 5 mM group; **P < 0.01, ***P < 0.001 versus the HG group. [file Image_2.jpeg]
